# The Effects of Propofol on a Human *in vitro* Blood-Brain Barrier Model

**DOI:** 10.3389/fncel.2022.835649

**Published:** 2022-05-11

**Authors:** Jason M. Hughes, Olivia R. Neese, Dylan D. Bieber, Kirsten A. Lewis, Layla M. Ahmadi, Dustin W. Parsons, Scott G. Canfield

**Affiliations:** ^1^Department of Anatomy, Cell Biology and Physiology, Indiana University School of Medicine, Terre Haute, IN, United States; ^2^Department of Biology, Indiana State University, Terre Haute, IN, United States

**Keywords:** blood-brain barrier, anesthesia toxicity, tight junction dysfunction, propofol, brain microvascular-like endothelial cells

## Abstract

**Background:**

Recently, the safety of repeated and lengthy anesthesia administration has been called into question, a subset of these animal studies demonstrated that anesthetics induced blood-brain barrier (BBB) dysfunction. The BBB is critical in protecting the brain parenchyma from the surrounding micro-vasculature. BBB breakdown and dysfunction has been observed in several neurodegenerative diseases and may contribute to both the initiation and the progression of the disease. In this study we utilize a human induced pluripotent stem cell (iPSC) derived-BBB model, exhibiting near *in vivo* properties, to evaluate the effects of anesthetics on critical barrier properties.

**Methods:**

iPSC-derived brain microvascular endothelial cells (BMECs) expressed near *in vivo* barrier tightness assessed by *trans-*endothelial electrical resistance and para-cellular permeability. Efflux transporter activity was determined by substrate transport in the presence of specific inhibitors. *Trans-*cellular transport was measured utilizing large fluorescently tagged dextran. Tight junction localization in BMECs was evaluated with fluorescent microscopy. The anesthetic, propofol was exposed to BMECs at varying durations and concentrations and BBB properties were monitored post-exposure.

**Results:**

Following propofol exposure, BMECs displayed reduced resistance and increased permeability indicative of a leaky barrier. Reduced barrier tightness and the dysregulation of occludin, a tight junction protein, were partly the result of an elevation in matrix metalloproteinase (MMP) levels. Efflux transporter activity and *trans-*cellular transport were unaffected by propofol exposure. Propofol induced barrier dysfunction was partially restored following matrix metalloproteinase inhibition.

**Conclusion:**

For the first time, we have demonstrated that propofol alters BBB integrity utilizing a human *in vitro* BBB model that displays key *in vivo* characteristics. A leaky BBB enables otherwise impermeable molecules such as pathogens and toxins the ability to reach vulnerable cell types of the brain parenchyma. A robust human *in vitro* BBB model will allow for the evaluation of several anesthetics at fluctuating clinical scenarios and to elucidate mechanisms with the goal of ultimately improving anesthesia safety.

## Introduction

The American Society of Anesthesiology estimates that millions of people undergo anesthesia each year in the United States. A substantial body of work has demonstrated that anesthesia, specifically at sustained or multiple exposures has cognitive and neurologic effects, primarily through neuron toxicity (Mason et al., 2010; Seubert et al., 2013; Acharya et al., 2015; Jiang et al., 2018). More recently, preclinical animal studies and a number of large population-based human studies presented limited associations between anesthesia exposure and negative outcomes in children (Ing and Brambrink, 2019). A subset of rodent studies have demonstrated that several anesthetics have detrimental effects on the blood-brain barrier (BBB) (Sharma et al., 2014; Zhao et al., 2014; Acharya et al., 2015; Cao et al., 2015). BBB breakdown and dysfunction are often associated with a variety of disease, including stroke, Alzheimer’s disease, HIV infection and brain tumors (Hirano and Matsui, 1975; Jellinger and Attems, 2006; Sweeney et al., 2018). Interestingly, the effects of anesthetics on the human BBB have not been previously studied.

The BBB is critical in maintaining homeostasis between the brain parenchyma and the microvasculature (Weiss et al., 2009). The BBB is comprised of the barrier forming brain microvascular endothelial cells (BMECs) supported by neurovascular unit (NVU) cell types: astrocytes, neurons, and pericytes. NVU cell types are critical in the development, maintenance and support of the barrier forming BMECs (Obermeier et al., 2013). BMECs provide a physical, transport and metabolic barrier due to the expression of tight junction proteins and active nutrient and efflux transporters. These properties allow BMECs to tightly regulate the movement of ions, molecules and cells between the blood and the brain.

Anesthetics, specifically, isoflurane, sevoflurane, and propofol have been demonstrated to alter tight junction expression in rodent BBB models (Sharma et al., 2014; Zhao et al., 2014; Acharya et al., 2015; Cao et al., 2015). The dysregulation of tight junction proteins can induce barrier leakiness and enable otherwise impermeable pathogens, toxins, molecules and cells to reach the brain parenchyma, potentially contributing to anesthesia-induced damage (Acharya et al., 2015). Several cellular mechanisms have been proposed in animal models including: vascular endothelial growth factor (VEGF), heat shock protein (HSP), matrix metalloproteinase (MMPs) and heat inducible factor-1α (HIF-1α) (Hu et al., 2014; Sharma et al., 2014; Zhao et al., 2014), however, their role in human BBB degradation following anesthesia are unknown. A dysfunctional BBB may further exacerbate the actions of anesthetics or even increase the probability of developing a detrimental BBB-induced injury.

For the first time we investigated the detrimental effects of anesthesia on the human BBB. We hypothesize that clinically relevant anesthetics will diminish critical barrier properties of iPSC-derived BMECs. To evaluate and elucidate the cellular mechanisms of anesthesia on the human BBB we utilized BMECs derived from human induced pluripotent stem cells (iPSCs) (Stebbins et al., 2015; Canfield et al., 2017). iPSC-derived BMECs display several near *in vivo* like BBB properties including: elevated barrier integrity, expression of localized tight junction proteins, active efflux transporters and reduced transcellular transport. The enhanced BBB properties of iPSC-derived BMECs compared to other *in vitro* models in addition to their human origin enable them to screen the efficacy and safety of a number of anesthetics (Crone and Olesen, 1982).

## Materials and Methods

### Differentiation of Brain Microvascular Endothelial Cells

Brain microvascular endothelial cells (BMECs) were differentiated from human induced pluripotent stem cells (iPSCs) as previously described (Canfield et al., 2017). Briefly, iPSCs (IMR90, WiCell, Madison, WI, United States) between passage 30–55 were singularized using Accutase (Life Technologies, Carlsbad, CA, United States) and plated (1 × 10^5^) onto Matrigel (Corning Life Sciences, Corning, NY, United States) coated 6-well plates (Thermo Fisher Scientific, Waltham, MA, United States) and maintained in mTESR nutrient medium (STEMCELL Technologies, Vancouver, BC, Canada) at 37°C for 3 days. Following stem cell expansion, cells were treated with unconditioned medium (UM) consisting of Dulbecco’s Modified Eagle’s Medium/Ham’s F12 (DMEM-F12, Thermo Fisher Scientific, Waltham, MA, United States) supplemented with 20% Knockout Serum Replacement (Thermo Fisher Scientific, Waltham, MA, United States), 1 × minimum essential medium non-essential amino acids (Life Technologies, Carlsbad CA, United States), 1 mM L-glutamine (Life Technologies, Carlsbad, CA, United States), and 0.1 mM β-mercaptoethanol (Sigma-Aldrich, St. Louis, MO, United States) to initiate the differentiation. Cells were maintained in UM for 6 days at 37°C with daily media changes. Following UM treatment, the medium was switched to EC + / + consisting of human Endothelial Serum-Free Medium (hESFM, Life Technologies, Carlsbad, CA, United States) containing 20 ng/mL bFGF (STEMCELL), 1% platelet-poor plasma derived bovine serum (PDS, Thermo Fisher Scientific, Waltham, MA, United States) and 10 μM retinoic acid (RA, Sigma Aldrich, St. Louis, MO, United States) for 48 h. BMECs were plated onto their respective experimental platform and maintained in EC + / + with RA for 24 h at 37°C. Medium was then switched to EC ± media comprised of hESFM with 1% PDS for the duration of the experiment and BMECs comprised >99% of the cell types. Peak barrier properties are maintained for 4–7 days post sub-culture onto their respective experimental platforms. All barrier phenotyping is conducted within 4 days of sub-culture. To evaluate barrier integrity we utilized *Trans-*endothelial electrical resistance (TEER) and sodium fluorescein permeability. Tight junction localization and expression were verified with immunocytochemistry and western blot, respectively. Efflux transporter expression and activity were assessed with flow cytometry and the transport and accumulation of specific efflux substrates, respectively. Transcellular transport was monitored with dextran transport.

### Propofol Exposure

Following differentiation, IMR90-derived BMECs were seeded (10^6^ cells/cm^2^) onto Transwell filter inserts (Thermo Fisher Scientific, Waltham, MA, United States) coated with a collagen IV (Sigma Aldrich, St. Louis, MO, United States) fibronectin (Sigma Aldrich, St. Louis, MO, United States) matrix for *trans-*endothelial electrical resistance (TEER), efflux transporter activity, *trans-*cellular transport/accumulation and/or sodium fluorescein permeability assays. For efflux transporter accumulation 125,000 cells/cm^2^ were seeded onto matrigel coated 24 well plates (Thermo Fisher Scientific, Waltham, MA, United States). For immunostaining 10^6^ cells/cm^2^ were seeded onto matrigel coated 18 mm round cover slips (Thermo Fisher Scientific, Waltham, MA, United States). For western blot lysates, BMECs were seeded (10^4^ cells/cm^2^) onto matrigel coated 6 well plates. Following 24 h, media was transitioned to EC ± medium. BMECs were treated with either 0 (Control; DMSO), intralipid (Sigma) alone, or 10, 30, 50, or 100 μM propofol (Sigma Aldrich, St. Louis, MO, United States) in fresh EC ± medium (0.5 mL top, 1.5 mL bottom) on a rotational platform at 37°C for 3 h. Following propofol exposure media was replaced with EC ± medium and the respective experiments commenced.

### *Trans-*Endothelial Electrical Resistance

*Trans-*endothelial electrical resistance (TEER) was measured immediately prior to propofol exposure and 30 min, 1 h, 2 h, 3 h, and every 24 h following propofol exposure. TEER was monitored up to 96 h post-exposure. Electrical resistance was measured using an EVOM ohmmeter with STX2 electrodes (World Precision Instruments, Sarasota, FL, United States). TEER values are presented as Ω× cm^2^ following the subtraction of an unseeded Transwell insert and multiplication by 1.12 cm^2^ to account for surface area. Measurements were recorded immediately following removal of the samples from the incubator. Resistance was measured at least three independent times on each sample and from a minimum of three triplicate filter inserts for each experimental condition.

### Sodium Fluorescein Permeability

Sodium fluorescein (10 μM, 376 Daltons; Sigma Aldrich, St. Louis, MO, United States) was utilized to determine the permeability of the iPSC-derived BMEC barrier. Following 3 h of exposure to propofol, fresh EC medium ± was added to the Transwell system (0.5 mL top, 1.5 mL bottom) with EC medium ± containing 10 μM sodium fluorescein added to the top chamber and EC medium ± without sodium fluorescein added to the bottom chamber. The Transwell filter inserts were then placed back on the rotational platform at 37°C for 1 h. 150 μL aliquots were sampled from the bottom chamber at 15, 30, and 45 min and immediately replaced with pre-warmed EC ± medium. At 60 min, 150 μL aliquots were sampled from both the top and bottom chambers and fluorescence was recorded using a Synergy HTX Multi-Mode reader (BioTek, Winooski, VT, United States). Permeability coefficients were calculated based on the cleared volume of sodium fluorescein from the top chamber to the bottom chamber.

### Immunocytochemistry and Analysis of Tight Junction Localization

Immunocytochemistry was performed on 50 μM propofol treatment and non-treatment control groups. Primary antibody sources, dilutions and corresponding fixing agents are presented in Supplementary Table 1 After 3 h treatment with propofol, iPSC-BMECs were fixed in either cold 4% paraformaldehyde (diluted in PBS; Sigma Aldrich, St. Louis, MO, United States) or ice cold methanol (100%; Sigma Aldrich, St. Louis, MO, United States) for 15 min on a rocking platform at room temperature. Cells were blocked with 10% goat serum (diluted in PBS; Sigma Aldrich, St. Louis, MO, United States) for 1 h at room temperature on a rocking platform. Following blocking, cells were incubated in primary antibody (diluted in blocking solution) overnight at 4°C on a rocking platform. All secondary antibodies were diluted 1:200 in blocking solution. Cells were incubated for 1 h in secondary antibody solution on a rocking platform at room temperature, protected from light. Invitrogen ProLong™ Gold antifade reagent (Life Technologies, Carlsbad, NY, United States) was used in the preparation of slides. Images were taken on an Olympus PROVIS AX70 motorized fluorescent microscope (Olympus, Center Valley, PA, United States) fitted with a SPOT Pursuit USB camera (SPOT Imaging, Sterling Heights, MI, United States). Following immunostaining with claudin-5, occludin, and zonula occludens (ZO-1), discontinuous tight junctions were quantified and processed in Image J with a minimum of 10 fields containing approximately 30 cells/field from three separate differentiations. Area fraction index was calculated using the same images to determine the area of each image that displayed claudin-5, occludin, or ZO-1 immunoreactivity (Canfield et al., 2017).

### Western Blot

Following 50 μM propofol exposure, BMECs were washed three times with cold phosphate-buffered saline (PBS) and lysed using ice cold Pierce™ RIPA buffer (Thermo Fisher Scientific, Waltham, MA, United States) with Halt™ Protease and Phosphatase Inhibitor Cocktail (Thermo Fisher Scientific, Waltham, MA, United States). Cell lysates were quantified for total protein concentration using a Pierce™ Rapid Gold BCA Protein assay kit (Thermo Fisher Scientific, Waltham, MA, United States). Gels were run at 120 V for 1 h (12% precast gel; Claudin-5, Occludin, β-actin) or for 70 min (4–15% gels; ZO-1) in Tris/Glycine sodium dodecyl sulfate buffer using a Bio-Rad Mini-Protean^®^ Tetra Vertical Electrophoresis Cell (Bio-Rad, Hercules, CA, United States). After samples were separated, the protein was transferred to Bio-Rad Immun-Blot^®^ PVDF membranes (Bio-Rad,Hercules, CA, United States) at 100 V for 1 h in transfer buffer (Tris/Glycine with 20% methanol). The membranes were washed one time with Tris-buffered saline containing 0.1% Tween 20 (TBST) and blocked for 1 h in blocking solution (5% non-fat dry milk dissolved in TBST) at room temperature on a rocking platform. Membranes were then probed with primary antibodies (Supplementary Table 2) in 10 mL of blocking solution at 4°C overnight on a rocking platform. Membranes were washed three times with TBST at room temperature on a rocking platform for 10 min. Membranes were probed with horseradish peroxidase (HRP) conjugated secondary antibodies (Supplementary Table 2) in blocking solution for 1 h at room temperature on a rocking platform and then washed three additional times in TBST at room temperature on a rocking platform. Membranes were imaged using a LI-COR C-DiGit^®^ Blot Scanner (LI-COR, Lincoln, NE, United States) and images were quantified using ImageJ software version 1.52a.

### Efflux Transporter Activity

#### Accumulation Assay

Three common efflux transporters were investigated: p-glycoprotein (Pgp), multi-drug resistance protein (MRP-1) and breast cancer resistance protein (BCRP). BMECs were treated with 50 μM propofol (Sigma Aldrich, St. Louis, MO, United States) in EC ± buffer at 37°C on a rotational platform for 3 h. Following propofol exposure, media was exchanged with EC ± buffer ± inhibitor. KO143 (10 μM, Sigma Aldrich, St. Louis, MO, United States) served as a BCRP inhibitor, MK157 (10 μM, Sigma Aldrich, St. Louis, MO, United States) served as a MRP-1 inhibitor, and Cyclosporin A (10 μM, Sigma Aldrich, St. Louis, MO, United States) served as a Pgp inhibitor. The plate was placed on a rotational platform in a 37°C incubator for 30 min. Media was exchanged with EC ± buffer ± inhibitor and substrate and the plate was placed on a rotational platform in a 37°C incubator for 1 h. Hoechst (5 μM, Sigma Aldrich, St. Louis, MO, United States) served as the BCRP substrate, DCFDA (20 μM, Thermo Fisher Scientific, Waltham, MA, United States) served as a MRP-1 substrate, and Rhodamine 123 (10 μM, Sigma Aldrich, St. Louis, MO, United States) served as the Pgp substrate. Following substrate exposure, each well was rinsed twice with cold Dulbecco’s Phosphate Buffered Saline (Sigma Aldrich, St. Louis, MO, United States). Subsequently, 300 μl of RIPA assay buffer was added to each well. The plate was protected from light and placed on a rotational platform at 23°C for 10 min. The fluorescent intensity of the plate was measured using a Synergy HTX Multi-Mode reader.

#### Transporter Assay

Brain microvascular endothelial cells were exposed to propofol (50 μM; Sigma Aldrich, St. Louis, MO, United States) for 3 h at 37°C. Subsequently, media was exchanged with EC ± buffer ± inhibitor (0.5 mL top, 1.5 mL bottom) and the plate was incubated at 37°C on a rotational platform for 30 min. The media on the top of the Transwell insert was replaced with a substrate ± inhibitor in EC ± buffer. The plate was incubated at 37°C for 1 h on a rotational platform. Following incubation, 150 μL was sampled from the bottom of each well and transferred to a 96-well plate (Thermo Fisher Scientific, Waltham, MA, United States). The fluorescent intensity of the plate was measured using a Synergy HTX Multi-Mode reader.

### Flow Cytometry

Following propofol (50 μM) exposure, cells were treated with Accutase at 37°C on a rotational platform for 30 min. Cells were fixed with 100% methanol and triturated briefly. Following fixation, BMECs were washed in PBS^–/–^ twice. BMECs were blocked in 10% donkey serum (Sigma Aldrich, St. Louis, MO, United States) in PBS^–/–^ for 1 h. Primary antibodies (Supplementary Table 1) were added to the suspension at appropriate dilutions. The microcentrifuge tubes were then vortexed before incubating overnight on a rotational platform at 4°C. Following two washes, BMECs were exposed to secondary antibodies (Supplementary Table 1) at the appropriate dilution in blocking solution for 30 min at 25°C on a rotational platform. Samples were vortexed once during the incubation period. Two washes were completed and after the final wash, the sample was resuspended in 400 μL of wash buffer. Samples were transferred to a 96-well round bottom plate (Thermo Fisher Scientific, Waltham, MA, United States) and read on the flow cytometer (Guava EasyCyte 8HT, Millipore Corporation, Burlington, MA, United States).

### Transcytosis

To determine the effects of propofol on transcellular movement in BMECs we utilized the transcytosis and accumulation of a 10 kDa dextran (Alexa Fluor 488, 10 μM; Sigma). BMECs displaying elevated and depressed barriers were utilized. To obtain BMECs with depressed barriers (200–400Ω× cm^2^) the same differentiation was utilized as above but without RA. Three hours following propofol treatment (50 μM) on transwell-seeded BMECs, dextran was diluted in EC ± and added to the apical side of the transwell. Following 4 h on a rotating platform at 37°C, media was collected from the basolateral side of the transwell and read on a Synergy HTX Multi-Mode reader (BioTek, Winooski, VT, United States), revealing the rate of transcytosis. BMECs were then rinsed three times in PBS and lysed with RIPA. Following trituration, the lysate was collected and quantified using a fluorescent plate reader, indicating the level of dextran accumulation. Data was presented following subtraction of transcytosis and accumulation of dextran at 4°C to account for para-cellular movement of dextran.

### Matrix Metalloproteinase Activity

Brain microvascular endothelial cells were treated with 50 μM propofol in EC ± buffer at 37°C on a rotational platform for 3 h. The supernate of cell culture media was collected and centrifuged for 10 min at 1,000 *g*, 4°C. The MMP-2/MMP-9 activities were determined by a fluorescence kit (SensoLyte^®^ Plus 520 MMP-2 and MMP-9 Assay Kit, Cat No. AS-72224, AS-72017; AnaSpec, Fremont, CA, United States) following the manufacturer’s instructions. Briefly, MMP-2 and MMP-9 were isolated using antibody-coated 96 well plates, which were provided in the assay kit. MMP-2/9 substrates were added to antibody-coated plates and incubated at room temperature for 24 and 16 h, respectively. Fluorescent intensity was measured using a Synergy HTX Multi-Mode reader (BioTek, Winooski, VT, United States) and data is presented as a percent change from control.

### Matrix Metalloproteinase Inhibition

Forty eight hours following seeding onto *trans-*wells, BMECs were treated with 25 μM GM-6001 MMP inhibitor (Thermo Fisher Scientific, Waltham, MA, United States) dissolved in EC medium (±) for 30 min at 37°C on a rotational platform. After 30 min of pre-treatment with GM-6001 inhibitor, media was aspirated and replaced with fresh EC medium (+PDS/-bFGF) containing 25 μM GM-6001 inhibitor (with or without 50 μM propofol) and incubated at 37°C for 3 h on a rotational platform. Propofol treatment was removed after 3 h of exposure and fluorescein permeability and tight junction analysis was conducted as previously described.

### Cell Viability

To determine cell viability, BMECs were treated with 10, 50, 100, or 1,000 μM propofol in EC ± media at 37°C on a rotational platform. Following 3 h of treatment, propofol was aspirated and replaced with 100 μL of EC ± media. A MTT Cell Viability Assay Kit (Biotium, Fremont, CA, United States) was utilized to determine cell viability following propofol exposure. Following manufacturer’s instructions, 10 μL of MTT solution was added to each well and the plate was incubated at 37°C for 2 h on a rotational platform. After 2 h of incubation, 200 μL of DMSO (Sigma Aldrich, St. Louis, MO, United States) was added to each well, triturating several times to dissolve the formazan salt. Absorbance was measured at 570 nm using a Synergy HTX Multi-Mode reader and normalized by subtracting background absorbance measured at 630 nm.

### Statistical Analysis

Data are presented as mean ± SD. Each experimental group consisted of iPSC-derived BMECs from at least three separate differentiations. For the statistical analyses, SigmaStat software (Systat Software, Inc., San Jose, CA, United States) was used. Statistical comparisons were performed using one-way analysis of variance (ANOVA) from the pooled data. Equal Variance and Shapiro-Wilk Normality Assumptions were satisfied for the residuals of each ANOVA model with a *p* value of 0.05 to reject. Within each condition, all pairwise comparisons were conducted within the ANOVA context using *post hoc* tests with the pooled variance estimate, followed by Holm-Sidak step down correction for multiple testing. Adjusted *p*-values displayed in the text. *P* < 0.05 was considered significant. The manuscript adheres to the applicable STROBE and ARRIVE guidelines.

## Results

### Effects of Propofol on Barrier Tightness and Passive Permeability in Human Stem Cell-Derived Brain Microvascular Endothelial Cells

**FIGURE 1:**
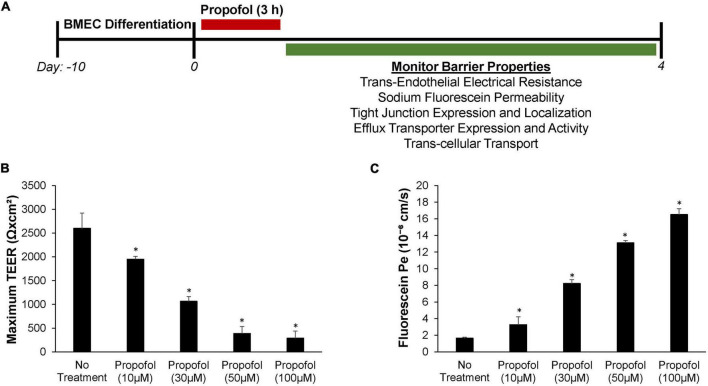
Effects of propofol on barrier tightness and passive permeability in human stem cell-derived brain microvascular endothelial cells (BMECs). **(A)** Following 3 h of propofol exposure several barrier properties were monitored in the BMEC population. **(B)** Maximum transendothelial electrical resistance (TEER) values were measured following treatment with propofol (0, 10, 30, 50, and 100 μM). **(C)** Sodium fluorescein permeability was measured 3 h following application of propofol (0, 10, 30, 50, and 100 μM). Permeability coefficients were calculated based on the cleared volume of sodium fluorescein from top chamber to bottom chamber. Statistical significance was calculated using ANOVA. *P < 0.05 verses no treatment. Values are presented as mean ± SD of three replicates from a single differentiation and experiments were repeated on three independent differentiations to verify statistical trends reported.

Forty eight hours after seeding, BMECs were treated with varying concentrations of propofol and barrier properties were evaluated (Figure 1A). To evaluate the effect of propofol on barrier tightness, TEER was measured immediately following propofol treatment and monitored every 24 h with maximum TEER values reported following treatment (Figure 1B). All concentrations of propofol evaluated significantly reduced TEER when compared to non-treatment control group (2,600 ± 321 Ω× cm^2^). A 10 μM propofol reduced the TEER of BMECs to 1,950 ± 61 Ω× cm^2^ (*p* < 0.05) and 30 μM lowered TEER to 1,065 ± 99 Ω× cm^2^ (*p* < 0.05), while 50 μM and 100 μM propofol had an even greater influence on reducing barrier tightness, with 50 μM reducing TEER to 388 ± 147 Ω× cm^2^ (*p* < 0.05) and 100 μM to 288 ± 148 Ω× cm^2^ (*p* < 0.05), respectively. To confirm propofol-induced decrease in TEER levels was not a result of decreased cell viability we measured cell viability following propofol treatments (10, 50, 100, 1,000 μM). Only a propofol concentration of 1 mM significantly reduced cell viability (Supplementary Figure 1). Additionally, to confirm that the propofol-induced decrease in barrier integrity was not due to an intralipid vehicle we measured TEER following control, intralipid alone, and intralipid with propofol (50 μM). Intralipid alone was indistinguishable from control (Supplementary Figure 2).

Sodium fluorescein assays were used to evaluate the effect propofol has on barrier permeability (Figure 1C). Immediately following propofol exposure, sodium fluorescein permeability significantly increased in iPSC derived BMECs with all concentrations surveyed when compared to non-treatment control group (*P*_*e*_ = 1.65 ± 0.11 × 10^–6^ cm/s). A 10 μM propofol elevated sodium fluorescein permeability to *P*_*e*_ = 3.28 ± 0.95 × 10^–6^ cm/s (*p* < 0.05) and 30 μM increased the permeability to fluorescein even further to *P*_*e*_ = 8.23 ± 0.45 × 10^–6^ cm/s (*p* < 0.05). While 50 μM propofol had a greater influence on fluorescein permeability than the latter with *P*_*e*_ = 13.11 ± 0.27 × 10^–6^ cm/s (*p* < 0.05), additionally 100 μM propofol had a significant effect on permeability with *P*_*e*_ = 16.52 ± 0.69 × 10^–6^ cm/s (*p* < 0.05).

Sodium fluorescein permeability was also used to evaluate the long term effects of propofol exposure on barrier integrity. Propofol treatments of 50 and 100 μM had significant effects on barrier permeability immediately following propofol treatment (6 h) and up to 4 days post-treatment (Table 1). Non-treatment BMECs exhibited a sodium fluorescein permeability of *P*_*e*_ = 1.62 ± 0.22 × 10^–6^ cm/s. BMECs treated with 50 μM propofol resulted in an elevated *P*_*e*_ of 6.44 ± 0.92 × 10^–6^ cm/s (*p* < 0.05) while treatment with 100 μM propofol further increased sodium fluorescein permeability to *P*_*e*_ = 13.84 ± 2.19 × 10^–6^ cm/s (*p* < 0.05). Treatment of BMECs with 10 μM propofol (*P*_*e*_ of 2.25 ± 0.53 × 10^–6^ cm/s) had no significant effect on sodium fluorescein permeability when compared to non-treatment BMECs. BMECs treated with propofol (50, 100 μM) exhibited an increased sodium fluorescein at 48 h [4.17 ± 0.04 × 10^–6^ cm/s, 7.16 ± 0.90 × 10^–6^ cm/s vs. no treatment (1.57 ± 0.22 × 10^–6^ cm/s)]. At 96 h post-propofol (50 and 100 μM) continued to display a weakened barrier [2.03 ± 0.31 × 10^–6^ cm/s and 7.15 ± 1.34 × 10^–6^ cm/s vs. no treatment (0.89 ± 0.14 × 10^–6^ cm/s)].

**TABLE 1:** Barrier integrity was evaluated 3, 48, and 96 h following propofol exposure.

Time (Post-treatment)	No treatment	Propofol (10 μM)	Propofol (50 μM)	Propofol (100 pM)
3 h	1.62 ± 0.22	2.25 ± 0.53	6.44 ± 0.92*	13.84 ± 2.19*
48 h	1.57 ± 0.22	0.95 ± 0.04	4.17 ± 0.04*	7.16 ± 0.90*
96 h	0.89 ± 0.14	0.98 ± 0.33	2.03 ± 0.31*	7.15 ± 1.34*

Fluorescein permeability coefficients (10^–^6 cm/s) were calculated in BMECs following propofol exposure (0 h/No treatment, 10, 50, 100 μM). Statistical significance was calculated using ANOVA at each time point. *P < 0.05 verses no treatment. Values are presented as mean ± SD of three differentiations.

### Propofol Dysregulates Occludin Protein Localization in Induced Pluripotent Stem Cell Derived Brain Microvascular Endothelial Cells

**FIGURE 2:**
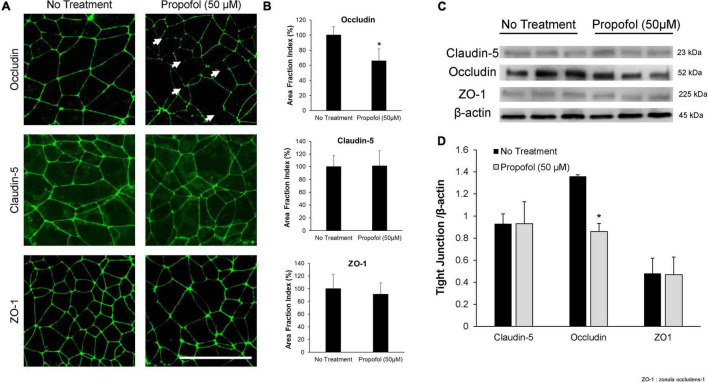
Analysis of tight junction continuity and expression in iPSCs derived BMECs following treatment with propofol. Tight junction protein localization and expression levels were examined 48 h following treatment with propofol (50 μM) for 3 h. **(A)** BMECs were immunocytochemically labeled for tight junction proteins: claudin-5, occludin, and ZO-1. Discontinuous tight junctions are indicated by white arrows. Scale bar = 50 μm. **(B)** Quantification of discontinuous tight junctions was performed by calculating the area of each image that displays claudin-5, occludin, or ZO-1 immunoreactivity, respectively (area fraction index). Values are presented as mean ± SD of three replicates from a single differentiation, and experiments were repeated on three independent differentiations to verify statistical trends reported. **(C)** Western blot of tight junction proteins following propofol treatment with β-actin loading control. Each lane represents a separate BMEC differentiation. **(D)** Quantification of western blots to compare tight junction protein expression levels. Propofol samples were independently normalized to each respective no-treatment sample. Statistical significance was calculated using Student’s *t*-test. *P < 0.05 versus no treatment. Values are presented as mean ± SD of three differentiations.

Tight junction protein expression and localization were evaluated in the diminished barrier properties in BMECs following exposure to propofol. Utilizing immunocytochemistry, tight junction localization was observed in BMECs 48 h following propofol treatment. Following immunochemistry of occludin, several discontinuous junctions (white arrows) were observed in propofol treated BMECs (Figure 2A). Area fraction index, an indicator of tight junction immunoreactivity, revealed that occludin levels decreased by 34 ± 16%compared to no-treatment (*p* < 0.05) (Figure 2B). Western blot analysis of tight junction proteins expression validated the diminished occludin immunoreactivity observed in propofol treated BMECs (Figure 2C). Cells treated with 50 μM propofol showed a 37% reduction in relative intensity of occludin expression when compared to non-treatment (*p* < 0.05) (Figure 2D). No significant changes were observed in expression or localization of claudin-5 and ZO-1.

### Brain Microvascular Endothelial Cell Efflux Transporters Activity Is Unaffected by Propofol Exposure

**FIGURE 3:**
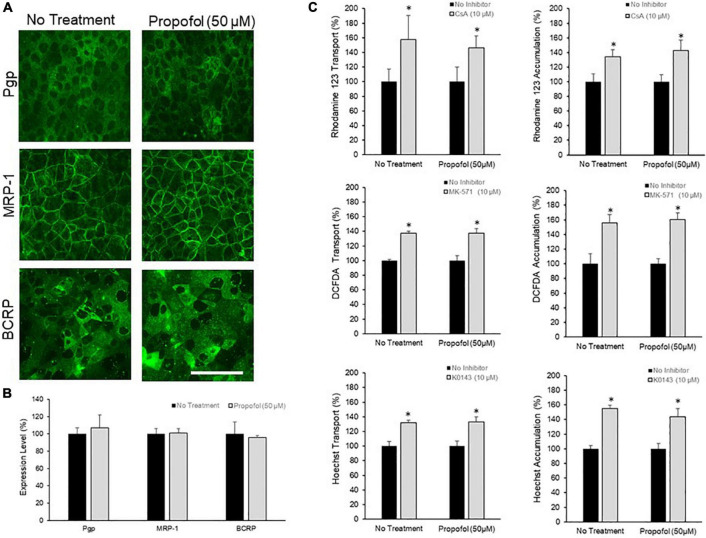
The effects of propofol on efflux transporters in BMECs. **(A)** iPSC-derived BMECs were immunolabeled for efflux transporters: Pgp, MRP-1, and BCRP following 3 h of propofol (50 μM) treatment. Scale bar = 100 μm. **(B)** Transporter expression levels were determined using flow cytometry. Geometric means of positively immunolabeled cell populations were analyzed to compare expression levels with and without propofol exposure. The data are normalized to no treatment expression levels. Statistical significant was determined using a Student’s *t*-test. Values are mean ± SD of three independent differentiations. **(C)** Efflux transporter activity was assessed by the transport of fluorescent substrates and optimized inhibitors (Pgp: Rhodamine/Cyclosporin A; MRP-1: DCFDA/MK571; BCRP: Hoechst/KO143) from the apical to the basolateral chamber in the two-compartment transwell model and the accumulation of substrate within the cells. Data is reported as a percentage change from no-inhibitor within each respective condition. Statistical significance was calculated using Student’s *t*-test. *P < 0.05 versus no inhibition. Values are presented as mean ± SD of three replicates from a single differentiation, and experiments were repeated on three independent differentiations to verify statistical trends reported.

The effects of 50 μM propofol on PgP, MRP-1, and BCRP expression and activity was investigated. Efflux transporter expression in BMECs was visualized by immunocytochemistry (Figure 3A) and quantified by flow cytometry (Figure 3B). Expression of PgP, MRP-1 and BCRP was unaffected by propofol exposure (Figure 3B). Efflux transporter activity was determined by measuring transport and accumulation of efflux transporter substrates (Figure 3C). There was no significant difference in Rhodamine 123 transport between control and propofol treatment conditions for PgP following inhibition with CsA (157.43 ± 33.39 and 145.99 ± 16.88). MRP-1 also showed an increased transport of DCFDA after inhibition with MK571 (137.11 ± 3.37 and 137.19 ± 6.28), but no significant difference between control and propofol groups. Likewise BCRP expressed in BMECs exhibited increased transport of Hoechst after exposure to the inhibitor KO143 (131.68 ± 3.49 and 133.33 ± 6.34) and no significant change between control and treatment groups. Accumulation of substrate was also investigated for PgP, MRP-1 and BCRP. There was no significant difference between control and treatment group accumulation for Pgp, MRP-1 or BCRP following inhibition.

### Propofol Does Not Affect Cellular Transcytosis in Brain Microvascular Endothelial Cells

**FIGURE 4:**
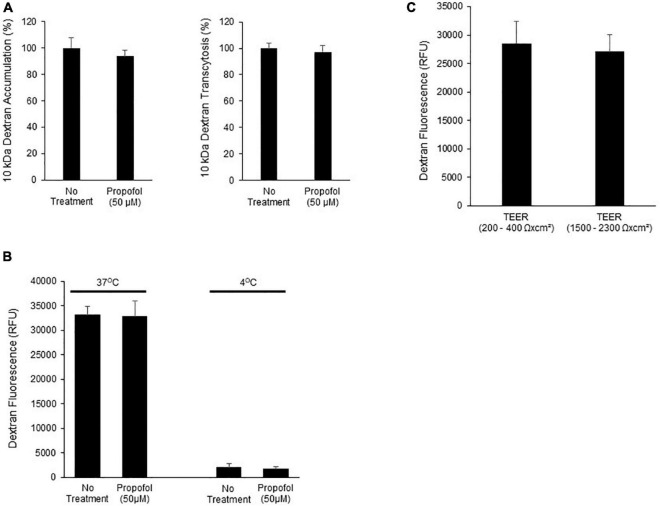
Determination of the effects of propofol on the ability of a fluorescently labeled dextran to cross BMECs. **(A)** BMECs were treated with propofol (50 μM) for 3 h prior to being presented with a fluorescently tagged dextran for 2 h. Fluorescently tagged dextran was measured from the bottom chamber (transcytosis) and within the BMEC population (accumulation). Raw fluorescence units are normalized to control BMECs. **(B)** To account for membrane fluidity in the ability of dextran to cross BMECs we conducted the assays at both 37° and 4°C. **(C)** To validate that dextran transcytosis was not related to changes in barrier tightness we evaluated the ability of dextran to cross BMECs with low TEER values (200–400 Ωxcm^2^) and high TEER values (1,500–2,300 Ωxcm^2^). Statistical significance was calculated using Student’s *t*-test. All experimental comparisons displayed no significance. Values are presented as mean ± SD of three replicates from a single differentiation, and experiments were repeated on three independent differentiations to verify statistical trends reported.

To determine the effects of propofol on uptake and transcytosis of a large molecule, a 10 kDa Alexa-Fluor tagged dextran was utilized. Following propofol treatment BMECs were treated with a fluorescently tagged dextran. The tagged dextran was quantified both within the cell (accumulation) and in the chamber below the BMEC-seeded transwell (transcytosis) and compared to non-treated BMECs. Following propofol treatment, BMECs had similar levels of accumulation and transcytosed dextran (94 ± 5%, 97 ± 5%; respectively; n.s.) compared to no treatment (100 ± 8, 100 ± 8%; respectively) (Figure 4A). When 10 kDa dextran transcytosis was conducted at 4°C, vesicular transport was significantly reduced (Figure 4B). Additionally, BMECs with TEER values of 200–400 Ω× cm^2^ had a similar level of dextran transcytosis compared to BMECs with TEER values ranging from 1,500 to 2,300 Ω× cm^2^ (Figure 4C) indicating that 10 kDa dextran transcytosis is occurring via a transcellular route and not a para-cellular route.

### Propofol-Induced Blood-Brain Barrier Damage Is Restored Following Matrix Metalloproteinase Inhibition

**FIGURE 5:**
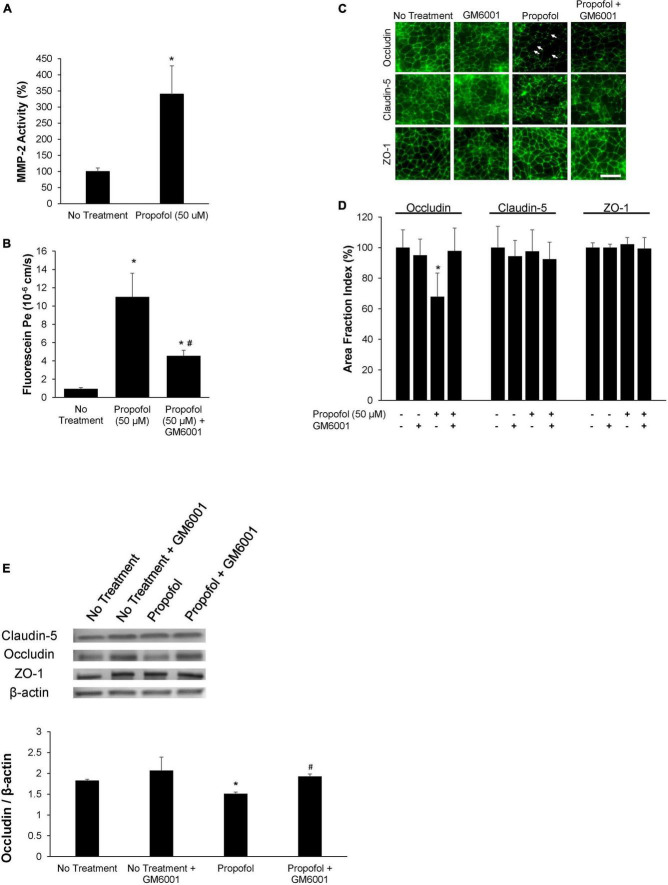
Propofol-induced matrix metalloproteinase-2 (MMP-2) partially diminishes barrier integrity. **(A)** Following 3 h of propofol exposure in BMECs, culture medium was collected and MMP-2 activity was analyzed. Data is reported as a percentage change from no treatment. Statistical significance was calculated using Student’s *t*-test. **(B)** An MMP-inhibitor, GM6001, was administered during propofol exposure. Following MMP-inhibition barrier integrity was assessed with fluorescein permeability. Permeability coefficients were calculated based on the cleared volume of sodium fluorescein from top chamber to bottom chamber. **(C)** Tight junction protein localization and expression levels were examined 48 h following treatment with either propofol, GM6001, or propofol and GM6001. BMECs were immunocytochemically labeled for tight junction proteins: claudin-5, occludin, and ZO-1. Discontinuous tight junctions are indicated by white arrows. Scale bar = 50 μm. **(D)** Quantification of discontinuous tight junctions was performed by calculating the area of each image that displays claudin-5, occludin, or ZO-1 immunoreactivity, respectively (area fraction index). **(E)** Western blot of tight junction proteins following propofol treatment with or without GM6001. Quantification of tight junction protein, occludin following normalization to β-actin. Statistical significance was calculated using ANOVA. *P < 0.05 verses no treatment; #*p* < 0.05 verses Propofol. Values are presented as mean ± SD of six differentiations.

The role of MMPs in propofol-induced BBB damage was investigated by utilizing a sodium fluorescein tracer in the presence of MMP inhibitor, GM6001. MMP2 and 9 activity were assessed after exposure to 50 μM propofol. Following propofol, BMECs had a 247 ± 88% increase in MMP2 activity compared to non-treatment (Figure 5A). Interestingly, propofol did not affect MMP 9 activity (increase of 4 ± 16% from control) (Supplementary Figure 3). To determine the role of MMP-2 induced barrier leakiness, BMECs were pretreated with an MMP inhibitor, GM 6001 (25 μM) for 30 min prior to and during propofol treatment (Figure 5B). Propofol (50 μM) increased sodium fluorescein permeability (11 ± 2.6 × 10^–^6 cm/s) compared to no-treatment control (0.9 ± 0.13 × 10^–^6 cm/s). GM6001 inhibition significantly attenuated the propofol-induced sodium fluorescein permeability increase observed following propofol treatment (4.5 ± 0.6 × 10^–^6 cm/s; *p* < 0.05 versus propofol). Additionally, GM6001 inhibition attenuated a propofol-induced decrease in TEER [decrease of 54 ± 8% vs. a decrease of 87 ± 2% compared to no-treatment (*p* < 0.05)] (Supplementary Figure 4). Additionally, the effects of GM6001 on tight junction localization was visualized with immunohistochemistry (Figure 5C). Several discontinuous occludin junctions (white arrows) were observed in propofol treated BMECs, however, they were not observed when GM6001 was administered with propofol (Figure 5C). Area fraction index revealed that propofol-induced occludin levels decreased by 33 ± 15% compared to no-treatment (*p* < 0.05) but the co-administration of GM6001 and propofol restored occludin levels back to no-treatment levels (decrease 3 ± 15%; n.s.) (Figure 5D). Following visualization of tight junction localization we evaluated the effects of GM6001 on tight junction expression during propofol treatment. Similar to our previous results, occludin expression was significantly depressed following propofol treatment (decrease 0.313 ± 0.016). GM6001 administered during propofol exposure restored occludin expression back to no treatment levels (1.88 ± 0.09 vs. 1.83 ± 0.03; Figure 5E).

## Discussion

This study demonstrates that an anesthetic, propofol, can induce blood-brain barrier defects in a human stem cell-derived blood-brain barrier model. The unique advantage of the study presented here is that the barrier model utilized is of human origin with several near *in vivo* barrier phenotypes. Our major findings are summarized as follows: (1) Propofol significantly diminished BBB integrity as observed as a decrease in TEER and an increase in sodium fluorescein permeability. (2) Propofol diminished occludin expression and localization. (3) Propofol does not affect cell viability, efflux transporter expression or activity, or *trans-*cellular transport. (4) Propofol enhanced MMP-2 activity and inhibition of MMP activity in part reduced the propofol-induced barrier damage. In summary, propofol was detrimental to the integrity of the barrier but did not affect the active components (i.e., efflux transporters, *trans-*cellular transport). The damaging effects of propofol were in part mitigated by treating BMECs with a global MMP inhibitor prior to and during propofol exposure implicating that additional cellular mechanisms are responsible for barrier breakdown following propofol exposure.

Anesthetic agents are regularly used in a variety of medical procedures for individuals of all ages with very little known about the long-term effects on the brain (Dyer et al., 1995; Acharya et al., 2015; Disma et al., 2018; Olutoye et al., 2018). Rodent models have demonstrated that several anesthetics, both volatile and lipophilic, have detrimental effects on NVU populations and ultimately functional discrepancies later in life (Stratmann et al., 2009; Shen et al., 2013). Importantly, the anesthetic-induced neurotoxicity was specific to a limited brain development window often associated with a period of brain growth when neuronal populations are vulnerable (Jevtovic-Todorovic et al., 2003).

More recently, several human studies have demonstrated the negative effects observed in animal studies may not be as robust in the human population and illustrate a need for effective and competent *in vitro* human models to further evaluate the safety of anesthetics in the human population (O’Leary and Warner, 2017; Disma et al., 2018). A limited number of reports have investigated the effects of anesthetics on the structure and function of the BBB in a variety of models. The clinical significance of these studies must be carefully measured as interspecies differences in the BBB exist (Syvänen et al., 2009; Warren et al., 2009). The utilization of human primary or immortalized BBB models alleviate some of these concerns, however, suboptimal barrier phenotypes often limit the extent of their efficacy (Calabria and Shusta, 2008). The validity of iPSC-derived BBB models have been questioned due to a mixed endothelial: epithelial transcriptional profile (Lu et al., 2021); yet, these iPSC-derived like BMECs exhibit near *in vivo* barrier function, an essential component of a BBB model (Workman and Svendsen, 2020). More recently, iPSC-derived BMECs have been utilized to enhance tissue engineering models, replicate pathological conditions, and unveil novel therapeutic approaches in the BBB (Li et al., 2021; Neal et al., 2021; Noorani et al., 2021; Raut et al., 2021; Wellens et al., 2021; Wu et al., 2021). Three common anesthetics, propofol, isoflurane, and sevoflurane, have independently demonstrated a degree of BBB damage (Sharma et al., 2014; Zhao et al., 2014; Acharya et al., 2015). Interestingly, propofol induces apoptosis in neuronal populations by potentially effecting astrocyte-derived brain derived neurotrophic factor (Liu et al., 2017). However, propofol does not appear to induce apoptosis in astrocytes and very little is known about the effects of propofol on pericytes, another critical cell type of the NVU (Yan et al., 2017). Due to the role of NVU cell types in the support, maintenance and the development of barrier forming BMECs any propofol-induced injuries could have a direct effect on barrier properties.

Propofol is an anesthetic agent commonly used for both the induction and maintenance of anesthesia in both short-term procedures and long-term sedation. Propofol similarly to the volatile anesthetics, isoflurane and sevoflurane, enhance GABA transmission but is administered intravenously compared to inhalation (Kim et al., 2018). Exposure of iPSC-derived BMECs to isoflurane and sevoflurane did not alter barrier integrity (data not shown). Determining an *in vivo* like concentration is challenging as propofol readily binds red-blood cells and circulating plasma proteins (Altmayer et al., 1995). However, propofol is lipophilic and the brain concentration is believed to be much higher compared to peripheral tissues (Riu et al., 2000). Representative *in vitro* concentrations have been reported to be as low as 3 μM and as high as 50 μM (Sall et al., 2012; Long et al., 2017). We observed a small reduction in barrier tightness (decreased TEER and increased fluorescein permeability) following 10 μM propofol administration, however, within 3 h barrier integrity (Table 1) was indistinguishable from no treatment implicating that barrier effects of propofol was not sustained. Additionally, tight junction localization was unchanged following 10 μM propofol. A robust barrier loss was observed following exposure to 50 μM propofol and was within clinical limits thus all subsequent experiments were conducted at this concentration. Without barrier supporting NVU cell types, iPSC-derived BMEC barrier properties begin to diminish four to 7 days post sub-culture; limiting the extent of propofol-induced barrier dysfunction we are able to observe. However, these results implicate at least in part that the safety of propofol anesthesia should be further studied, specifically in terms of its action on the blood-brain barrier.

Several studies have demonstrated a strong correlation between junctional continuity and barrier phenotype (Persidsky et al., 2006). Isoflurane was previously demonstrated to decrease occludin expression in primary human brain microvascular endothelial cells (Zhao et al., 2014). Similarly, we observed a significant decrease in expression and localization of occludin along with a loss in barrier tightness following propofol exposure. Loss of occludin localization and expression are likely responsible for the observed barrier loss following propofol exposure. Additionally, we investigated the effects of propofol on the expression and activity of efflux transporters. Regev et al. demonstrated that the anesthetics: benzyl alcohol, non-aromatic chloroform, and diethyl ether abolished Pgp activity; however, these studies were conducted in non-brain-microvascular endothelial cells (Regev et al., 1999). We previously demonstrated that iPSC-derived BMECs express functional efflux transporters including: Pgp, MRP-1 and BCRP (Canfield et al., 2017). Propofol exposure did not have an effect on efflux transporter activity in iPSC-derived BMECs. It is difficult to benchmark these results to the literature as there are no previous studies that have investigated the effects of propofol on efflux transporter activity or expression. Finally, we investigated non-specific transcytosis following propofol. Brain endothelium has a significantly reduced rate of transcytosis compared to peripheral endothelial cells (Daneman et al., 2010). Isoflurane has been demonstrated to increase caveolar-dependent transcytosis, however, propofol exposure did not alter non-specific transcytosis in iPSC-derived BMECs (Spieth et al., 2021).

Anesthetic-induced barrier damage mediators have been previously investigated, including the roles of reactive oxygen species, vascular endothelial growth factor, heat-inducible factor-1α, and matrix metalloproteinase (Sharma et al., 2014; Zhao et al., 2014). Previously, MMPs have been demonstrated to de-localize tight junctions and digest basement membrane proteins contributing to an increase in barrier leakiness (Feng et al., 2011). Specifically, MMP-2 and MMP-9 have been implicated in anesthetic-induced blood-brain barrier breakdown (Hu et al., 2014). MMP-9 activity is often associated with an increase in VEGF-induced BBB permeability (Valable et al., 2005). Human iPSC-derived BMECs had elevated MMP-2 activity following propofol exposure; however, MMP-9 activity and VEGF expression (data not shown) remained unchanged. The addition of a global MMP inhibitor during propofol exposure partially protected barrier tightness in iPSC-derived BMECs. These data indicate that other non-MMP signaling mechanisms either independently or in unison with MMP-2 contribute to propofol-induced barrier breakdown.

Finally, to our knowledge, we are the first to investigate the toxic effects of propofol on a relevant human BBB model. Similarly to models of different sources, we observed propofol diminishing barrier integrity by decreasing tight junction expression and localization. These actions were in part mitigated with the addition of a global MMP inhibitor. The utilization of a human iPSC-derived BBB model with robust *in vivo* like properties demonstrates that further studies are warranted in understanding the effects of anesthetics on the blood-brain barrier both acutely and long-term. Specifically, a better understanding of cellular mechanisms involved in anesthetic-induced BBB breakdown would unveil novel therapeutic interventions to further enhance anesthesia safety.

## Data Availability Statement

The raw data supporting the conclusions of this article will be made available by the authors, without undue reservation.

## Author Contributions

JH, ON, and SC designed and conducted the experiments, analysis, interpretation, and wrote the manuscript. DB, KL, LA, and DP conducted the experiments, data analysis, and interpretation. All authors contributed to the article and approved the submitted version.

## Conflict of Interest

The authors declare that the research was conducted in the absence of any commercial or financial relationships that could be construed as a potential conflict of interest.

## Publisher’s Note

All claims expressed in this article are solely those of the authors and do not necessarily represent those of their affiliated organizations, or those of the publisher, the editors and the reviewers. Any product that may be evaluated in this article, or claim that may be made by its manufacturer, is not guaranteed or endorsed by the publisher.

## Supplementary Material

Supplementary Figure 1 Cell viability was assessed in BMECs following propofol exposure. Following propofol exposure cell viability was determined utilizing a MTT cell viability assay kit. Data is reported as a percentage change from control (no Propofol). Statistical significance was calculated using ANOVA. **P* < 0.05 versus control. Values are presented as mean ± SD of three differentiations.

Supplementary Figure 2 Barrier integrity was evaluated following intralipid exposure. BMECs were exposed to DMSO (control solvent), intralipid alone, and propofol with intralipid. Data is reported as a percentage change from control. Statistical significance was calculated using ANOVA. Values are presented as mean ± SD of three differentiations.

Supplementary Figure 3 MMP-9 activity was assessed in BMECs following propofol exposure. Following 3 h of propofol exposure in BMECs, culture medium was collected and MMP-9 activity was analyzed. Data is reported as a percentage change from no treatment. Statistical significance was calculated using Student’s *t*-test. Values are presented as mean ± SD of three replicates from a single differentiation, and experiments were repeated on three independent differentiations to verify statistical trends reported.

Supplementary Figure 4 Propofol-induced matrix metalloproteinase-2 (MMP-2) partially diminishes *trans-*endothelial electrical resistance. An MMP-inhibitor, GM6001, was administered during propofol exposure. Following MMP-inhibition barrier integrity was assessed with *trans-*endothelial electrical resistance (TEER). TEER was normalized to a percentage change from no-treatment. Statistical significance was calculated using ANOVA. **P* < 0.05 verses no treatment; #*p* < 0.05 verses Propofol. Values are presented as mean ± SD of three differentiations.

Supplementary Table 1 Antibody information for immunocytochemistry assays.

Supplementary Table 2 Antibody information for western blot assays.

## Abbreviations

BBB: blood-brain barrier
BMECs: brain microvascular endothelial cells
NVU: neurovascular unit
HSP: heat shock protein
MMPs: matrix metalloproteinase
HIF-1α: heat inducible factor-1 α
iPSCs: Human induced pluripotent stem cells
hESFM: human endothelial serum-free medium
bFGF: basic fibroblast growth factor
PDS: platelet-poor plasma derived bovine serum
RA: retinoic acid
TEER: *Trans-*endothelial electrical resistance
ZO-1: zonula occludens
PBS: phosphate-buffered saline
TBST: Tris-buffered saline containing 0.1% Tween 20
HRP: horseradish peroxidase
Pgp: P-glycoprotein
MRP-1: multi-drug resistance protein
BCRP: breast cancer resistance protein
CsA: Cyclosporin A
DCFDA: dichlorodihydrofluorescein diacetate
MTT: 3-(4,5-dimethylthiazol-2-Yl)-2,5-diphenyltetrazolium bromide
DMSO: dimethyl sulfoxide
ANOVA: analysis of variance.
