# Supplementary material for: The Effects of Propofol on a Human in vitro Blood-Brain Barrier Model
Source: Front Cell Neurosci. 2022 May 11;16:835649. doi: 10.3389/fncel.2022.835649 (PMC9132176; doi:10.3389/fncel.2022.835649)
Supplement: Supplementary Figure 1 — Cell viability was assessed in BMECs following propofol exposure. Following propofol exposure cell viability was determined utilizing a MTT cell viability assay kit. Data is reported as a percentage change from control (no Propofol). Statistical significance was calculated using ANOVA. *P < 0.05 versus control. Values are presented as mean ± SD of three differentiations. [file Data_Sheet_1.PDF]

Supplementary Figure 1

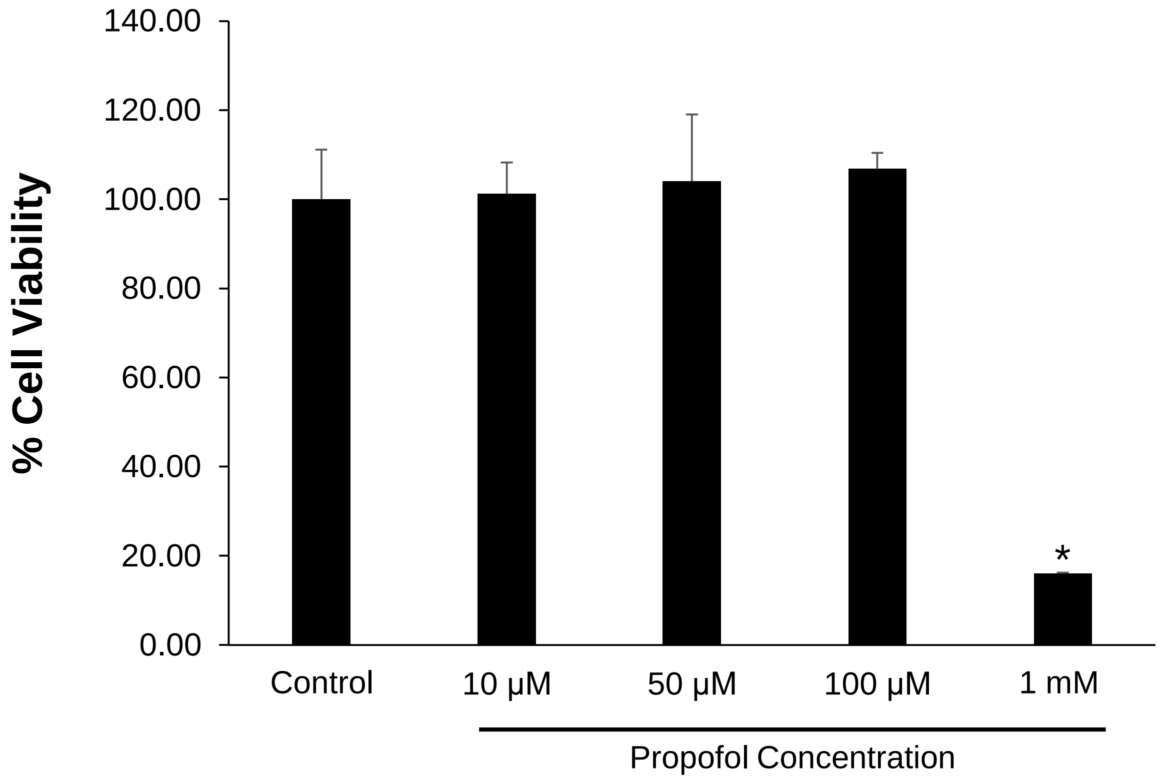

Supplementary Figure 2

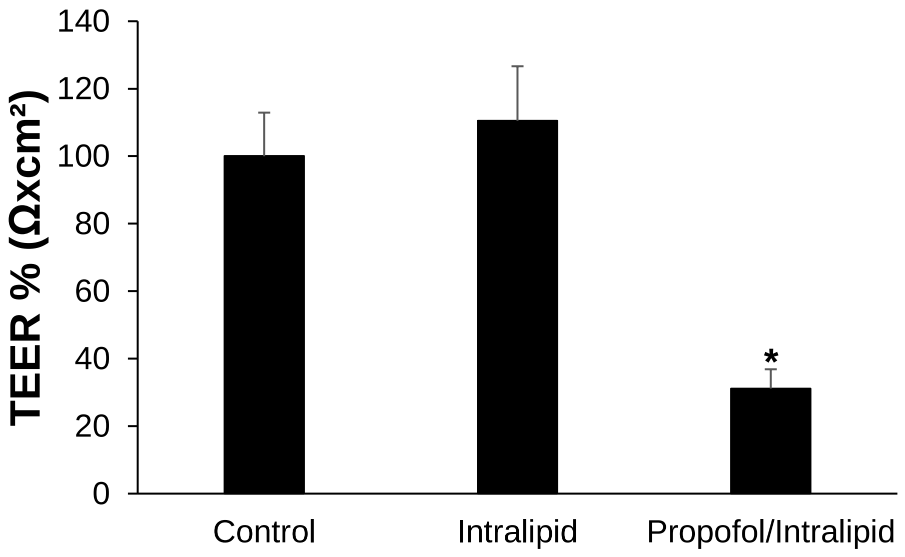

Supplementary Figure 3

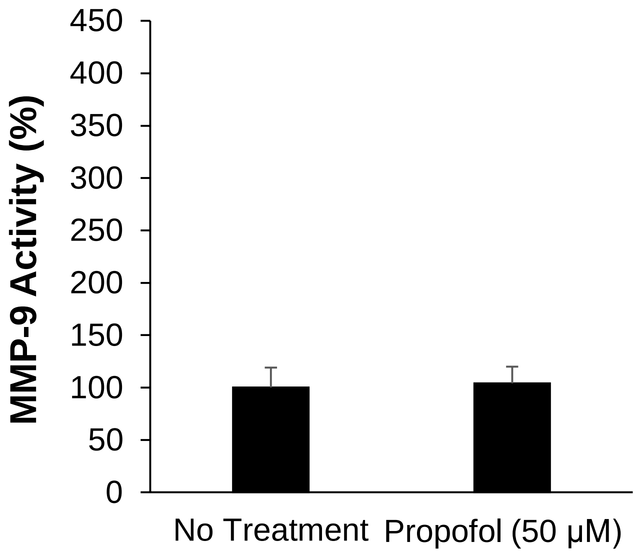

Supplementary Figure 4

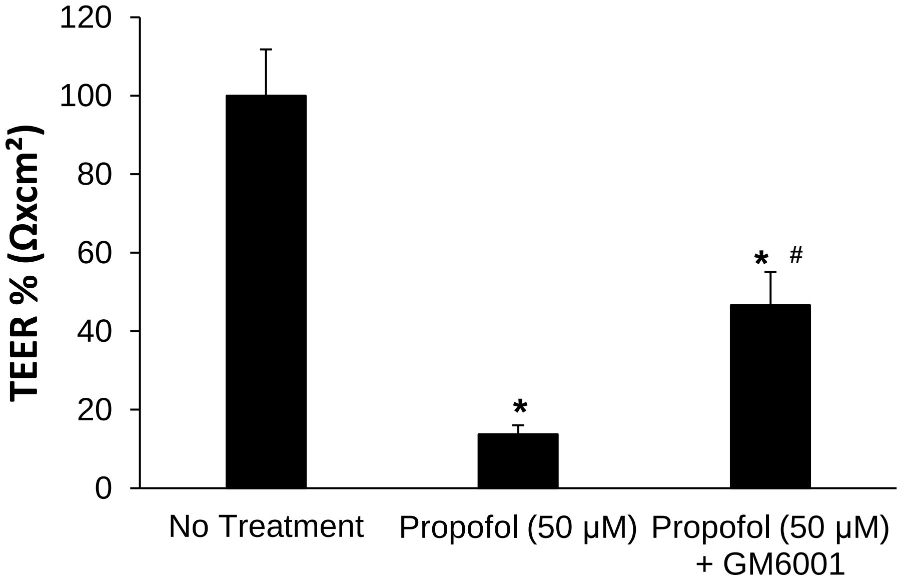

Supplementary Table 1

Primary Antibody	Vendor, clone or product #, Species	Fixative	Dilution	Block	Secondary Antibody
Claudin-5	ThermoFisher, clone 4C3C2, Mouse IgG1 monoclonal	MeOH	1:50	10% PBSG	Goat anti-Mouse IgG (H + L) Secondary Antibody, Alexa Fluor® 488 conjugate (ThermoFisher, A11001)
Occludin	ThermoFisher, clone OC-3F10, Mouse IgG1 monoclonal	MeOH	1:200	10% PBSG	Goat anti-Mouse IgG (H + L) Secondary Antibody, Alexa Fluor® 488 conjugate (ThermoFisher, A11001)
ZO-1	ThermoFisher, clone ZO1-1A12, Mouse IgG1 monoclonal	MeOH	1:100	10% PBSG	Goat anti-Mouse IgG (H + L) Secondary Antibody, Alexa Fluor® 488 conjugate (ThermoFisher, A11001)
GLUT-1	ThermoFisher, clone SPM498, Mouse IgG2a-kappa monoclonal	MeOH	1:500	10% PBSG	Goat anti-Mouse IgG (H + L) Secondary Antibody, Alexa Fluor® 488 conjugate (ThermoFisher, A11001)
PECAM-1	ThermoFisher, clone CD31/PECAM-1, Rabbit polyclonal	MeOH	1:25	10% PBSG	Goat anti-Rabbit IgG (H + L) Secondary Antibody, Alexa Fluor® 488 conjugate (ThermoFisher, A11034)
P-gp	ThermoFisher, clone C219, Mouse IgG2a monoclonal	MeOH	1:25	10% PBSG	Goat anti-Mouse IgG (H + L) Secondary Antibody, Alexa Fluor® 488 conjugate (ThermoFisher, A11001)
BCRP	Millipore, clone 5D3, Mouse IgG2b-kappa monoclonal	4% PFA	1:50	10% PBSG	Goat anti-Mouse IgG (H + L) Secondary Antibody, Alexa Fluor® 488 conjugate (ThermoFisher, A11001)
MRP1	Millipore, clone QCRL-1, Mouse IgG1 monoclonal	MeOH	1:25	40% PBSG	Goat anti-Mouse IgG (H + L) Secondary Antibody, Alexa Fluor® 488 conjugate (ThermoFisher, A11001)

Supplementary Table 2

Primary Antibody	Vendor, clone, and Species	Dilution	Block	Secondary Antibody	Dilution	Block	Band Size (M.W.)
Claudin-5	ThermoFisher, clone 4C3C2, Mouse IgG1 monoclonal	1:250	5% non-fat dry milk in TBST	HRP-conjugated ThermoFisher, cat # PA1-74421, Goat IgG polyclonal (Mouse IgG1 paraproteins)	1:2000	5% non-fat dry milk in TBST	23 kDa
Occludin	ThermoFisher, clone OC-3F10, Mouse IgG1 monoclonal	1:500	5% non-fat dry milk in TBST	HRP-conjugated ThermoFisher, cat # PA1-74421, Goat IgG polyclonal (Mouse IgG1 paraproteins)	1:2000	5% non-fat dry milk in TBST	52 kDa
ZO-1	ThermoFisher, clone ZO1-1A12, Mouse IgG1 monoclonal	1:100	5% non-fat dry milk in TBST	HRP-conjugated ThermoFisher, cat # PA1-74421, Goat IgG polyclonal (Mouse IgG1 paraproteins)	1:2000	5% non-fat dry milk in TBST	225-230 kDa
β-actin	Cell Signaling Technology, clone 8H10D10, Mouse IgG2b monoclonal	1:1000	5% non-fat dry milk in TBST	HRP-conjugated Cell Signaling Technology, cat # 7076, Horse IgG anti-mouse IgG	1:5000	5% non-fat dry milk in TBST	45 kDa
